# A Narrative Review of Emerging Immune Targets in Neuroinflammation-Driven Epileptogenesis: From Complement Pathways to Immune Checkpoints

**DOI:** 10.7759/cureus.97750

**Published:** 2025-11-25

**Authors:** Meet Popatbhai Kachhadia, Rochelle Boguslavskiy, Olivia Mattner, Amy W Laitinen, Pooja Patel, Neil U. Shah, Usmaan Topiwala

**Affiliations:** 1 Neurology, Florida Atlantic University Charles E. Schmidt College of Medicine, Boca Raton, USA; 2 Neurology, Florida Atlantic University, Boca Raton, USA; 3 General Medicine, Smt. Nathiba Hargovandas Lakhmichand (NHL) Municipal Medical College, Ahmedabad, IND

**Keywords:** biomarkers, complement system, epileptogenesis, immune checkpoints, neuroinflammation

## Abstract

Epileptogenesis, the process by which a normal brain becomes epileptic, has long been attributed to excitotoxicity and neuronal hyperexcitability. Advances in neuroimmunology now indicate that neuroinflammation contributes materially to seizure initiation, propagation, and chronicity, opening avenues for therapies that aim to modify disease biology rather than merely suppress symptoms. To maintain a clear scope, this review focuses on three interconnected pathways with the strongest mechanistic and translational support: 1) the complement cascade-particularly C1q, C3/C3aR, and C5aR1, as mediators of maladaptive synaptic pruning and glia-neuron crosstalk; 2) cytokine signaling centered on interleukin (IL)-1β (and IL-6 where relevant) as convergent drivers of excitability and gliosis; and 3) programmed cell death protein 1 (PD-1)/programmed death-ligand 1 (PD-L1) checkpoint signaling within neurons and glia as an intrinsic brake on excitability and inflammation. Other mediators (e.g., tumor necrosis factor-α, cytotoxic T-lymphocyte antigen 4, T cell immunoreceptor with Ig and ITIM domains, lymphocyte activation gene 3, and the NLRP3 inflammasome) are discussed in brief to contextualize the landscape but are not primary foci unless data bear directly on clinical translation. Regarding causality, the evidence supports a contributory and, in defined contexts, causal role for inflammatory pathways in epileptogenesis. In autoimmune encephalitides (e.g., anti-N-methyl-D-aspartate receptor, anti-leucine-rich glioma-inactivated 1), seizure control frequently follows immunotherapy rather than ASMs alone, consistent with a causal immune mechanism in at least a subset of cases. In experimental systems, manipulating inflammatory nodes, blocking IL-1 signaling or toll-like receptor 4, inhibiting C5aR1, or attenuating BBB-driven transforming growth factor-beta signaling reduces epileptiform activity and epileptogenesis, strengthening biological plausibility. Nevertheless, causality is disease- and time-dependent; in many acquired epilepsies, neuroinflammation likely functions as a disease amplifier that sustains and worsens an epileptic network established by an initial insult rather than serving as the sole initiator. Within this framed scope, we synthesize recent experimental, translational, and clinical data on immune dysregulation in epilepsy. We emphasize mechanisms by which complement-mediated synaptic pruning, glial activation, and checkpoint dysfunction contribute to neuronal damage, circuit remodeling, and chronic seizure activity. We appraise current and investigational agents according to mechanism, efficacy signals, and clinical applicability (e.g., C5aR1 antagonism, IL-1 pathway blockade, and approaches that locally augment PD-1/PD-L1 signaling). We also review emerging biomarkers, including cerebrospinal fluid IL-1β, serum glial fibrillary acidic protein, and translocator protein positron-emission tomography, as promising but still developmental tools for stratifying patients and guiding immunotherapy, noting that validation for routine clinical selection is ongoing. Finally, we discuss combination strategies that pair innate and adaptive targets (e.g., NLRP3 inhibition with PD-L1 agonism) as exploratory avenues that may yield synergy while requiring careful infectious-risk mitigation and biomarker-guided deployment.

## Introduction and background

Epilepsy is one of the most prevalent and dangerous neurological disorders, affecting over 50 million people worldwide and characterized by recurrent, unprovoked seizures. Despite advances in antiseizure medications (ASMs), about one-third of individuals develop drug-resistant epilepsy (DRE) after appropriately chosen and tolerated ASM trials, underscoring the need for therapies that modify disease biology rather than merely suppress symptoms [1].

An initial brain insult like trauma, infection, stroke, genetic channelopathy, or prolonged seizures releases danger-associated molecular patterns from stressed cells; these signals activate innate immune receptors on microglia and astrocytes, lower seizure threshold, and, over months to years, drive persistent network changes that outlast the original trigger. Neuroinflammation has emerged as a key contributor to the development and progression of epileptogenesis, the gradual transformation of a previously healthy brain into one with an enduring predisposition to spontaneous seizures. Activated microglia and astrocytes, cytokines and chemokines, complement proteins, and infiltrating peripheral immune cells interact with blood-brain barrier (BBB) alterations to drive synaptic remodeling and network hyperexcitability. These processes are increasingly documented across experimental models and human epileptic tissue [2].

BBB tight-junctions are loosened by inflammatory enzymes, permitting albumin and leukocytes to enter the parenchyma; astrocytic uptake of albumin activates transforming growth factor-beta (TGF-β) signaling, downregulates Kir4.1 channels, impairs K⁺ buffering, and fosters excitatory synaptogenesis. In parallel, complement tag synapses for pruning and reactive glia reshape circuits, together creating a self-reinforcing, hyperexcitable network. Among innate and glial mediators, the interleukin (IL)-1β pathway, tumor necrosis factor-α (TNF-α), and high-mobility group box-1 (HMGB1) are particularly well-characterized. Convergent experimental and translational data indicate that these signals lower seizure threshold, potentiate neuronal injury, and promote network instability. In parallel, toll-like receptors (TLRs), notably TLR4, and the complement cascade facilitate proepileptogenic circuit reorganization and synaptic loss; overactivation of the classical complement pathway (C1q→C3) has been implicated in pathological synaptic pruning, with supportive observations in both models and human epileptic cortex [2,3].

IL-1β can enhance N-methyl-D-aspartate (NMDA) receptor-mediated Ca²⁺ influx; HMGB1 released from injured neurons signals via TLR4/receptor for advanced glycation end-products (RAGE) on glia; and complement components (C1q/C3) localize to the epileptic cortex, where they correlate with synaptic loss, together providing a coherent link between inflammation and excitability. Beyond classical cytokine and complement biology, immune checkpoint pathways are gaining attention for their immunoregulatory and neuromodulatory roles in the central nervous system (CNS). The programmed cell death protein 1 (PD-1)/programmed death-ligand 1 (PD-L1) axis is functionally expressed in neurons and glia and can directly modulate neuronal excitability and synaptic transmission; perturbation of this pathway alters plasticity in vivo. While cytotoxic T-lymphocyte antigen 4 (CTLA-4) remains primarily an immune-cell checkpoint with important CNS safety considerations (given neurological toxicities observed during systemic checkpoint blockade in oncology), both axes illustrate how checkpoint signaling might be harnessed to restore immune balance during epileptogenesis, albeit with caution and rigorous safety monitoring [2,4].

In principle, local augmentation of inhibitory checkpoint tone could dampen excitability and glial activation, but systemic manipulation must balance benefit against immune-related adverse events. Collectively, these insights have shifted the field from viewing neuroinflammation as a secondary by-product of seizures to recognizing it as a contributory driver of epileptogenesis in at least a subset of patients. This has opened therapeutic avenues that target upstream biology, such as IL-1 receptor blockade, complement inhibition (e.g., targeting C3 or C5aR1), and checkpoint pathway modulation, with the goal of disease modification beyond symptomatic seizure control. Early clinical signals (e.g., IL-1/IL-6 pathway agents in New Onset Refractory Status Epilepticus (NORSE)/febrile infection-related epilepsy syndrome (FIRES)) and robust preclinical data support continued translational work [2,5].

When evidence pertains specifically to adaptive autoimmunity (autoimmune-associated epilepsy), when findings generalize to innate/glial mechanisms across DRE (neuroinflammatory epileptogenesis), we state that scope explicitly, as both arms interact within the same patient [2].

Accordingly, this narrative review consolidates contemporary evidence on immune targets in neuroinflammation-driven epileptogenesis, with emphasis on the complement system, cytokine signaling, TLR pathways, and immune checkpoints. We integrate experimental and clinical data to appraise therapeutic promise, boundary conditions, and safety considerations for immunomodulation in epilepsy.

DRE and immune involvement

Epilepsy affects more than 50 million people worldwide and remains a major cause of neurological morbidity. Despite substantial advances in ASMs, about one-third of individuals develop DRE. Consistent with the International League Against Epilepsy consensus, DRE is defined as the failure of two appropriately chosen, tolerated, and correctly used ASM regimens, whether as monotherapies or in combination, to achieve sustained seizure freedom. This treatment-refractory group experiences higher risks of injury, cognitive decline, psychosocial impairment, and diminished quality of life, indicating that mechanisms beyond classical ion-channel dysfunction and neurotransmitter imbalance often sustain epileptogenesis [2,6].

Converging clinical and experimental data increasingly implicate immune dysregulation and neuroinflammation as central drivers of epileptogenesis, particularly in patients with persistent seizures despite ASM trials. Within this landscape, a practical distinction helps organize evidence and guide therapy. Autoimmune-associated epilepsy (AAE) denotes epilepsy in which adaptive immune mechanisms, often antibodies against neuronal surface proteins such as N-methyl-D-aspartate receptor (NMDAR), leucine-rich glioma-inactivated 1 (LGI1), CASPR2, or Gamma-Aminobutyric Acid-B, play a causative role; seizures in these disorders frequently respond incompletely to ASMs but improve after immunotherapy (e.g., corticosteroids, intravenous immunoglobulin, plasma exchange, and, when needed, rituximab or cyclophosphamide). Neuroinflammatory epileptogenesis refers to epilepsy characterized by predominant innate and glial pathways, including microglial and astrocytic activation, along with cytokines, chemokines, complement signaling, and BBB dysfunction, which may occur with or without detectable neural autoantibodies. In practice, these entities exist on a spectrum: autoimmune encephalitides can evolve into chronic epilepsy sustained by glial and innate cascades, whereas many “nonautoimmune” DRE cases demonstrate persistent inflammatory signatures in tissue and biofluids. The net effect is a blurring of lines at the bedside, and clinicians increasingly integrate clinical context, antibody testing, cerebrospinal fluid (CSF) studies, and occasionally surgical pathology to position a given patient along the autoimmune-inflammatory axis and to rationalize trials of immunomodulation [2,7].

Throughout this review, we explicitly identify when data derive from adaptive immune (AAE) mechanisms vs. innate/glial (neuroinflammatory) pathways to help the reader track which targets generalize across DRE and which are antibody-specific. Illustrative of the adaptive end of this spectrum, anti-NMDAR encephalitis is characterized by antibodies to the GluN1 (NR1) subunit and presents with psychiatric and cognitive symptoms, dyskinesias, autonomic instability, and seizures. Although ASMs are used, meaningful seizure control typically follows immunotherapy, and earlier treatment is associated with better outcomes. A similar pattern holds in anti-LGI1 encephalitis, where faciobrachial dystonic seizures and limbic features often respond only after timely immunotherapy, with multiple cohorts showing improved seizure outcomes when treatment is initiated promptly. These disorders provide strong evidence that immune mechanisms can be causal in seizure generation, not merely epiphenomena [2,8,9].

Despite these advances, there is no immune-based therapy yet approved specifically as a disease-modifier for epilepsy, and standardized protocols or predictive biomarkers for chronic, nonencephalitic epilepsy remain incomplete. This therapeutic gap, combined with the mounting evidence for immune involvement, motivates systematic investigation of immune pathways, including complement, cytokine signaling, microglial-astrocyte crosstalk, TLR/HMGB1 signaling, and immune checkpoints, as candidates for rational, mechanism-guided intervention in both autoimmune epilepsies and the broader DRE population where inflammation persists in the absence of classical antibody markers [2,9].

Neuroinflammatory epileptogenesis: a newer framework

Over the past decade, epilepsy has been reframed from a disorder explained solely by neuronal hyperexcitability to one profoundly shaped by chronic neuroinflammation. CNS insults such as traumatic brain injury, infection, status epilepticus, or stroke can initiate a cascade involving microglial and astrocytic activation, proinflammatory cytokines and chemokines, complement pathway engagement, and BBB disruption. Once established, these processes interact in a feedforward loop that promotes neuronal injury, synaptic reorganization biased toward excitation, and persistent network hyperexcitability biology that can underpin both seizure recurrence and ASM refractoriness. Adenosine triphosphate (ATP), HMGB1, and extracellular DNA activate TLRs and RAGE, while BBB breakdown permits albumin and leukocytes to enter the parenchyma; albumin-triggered astrocytic TGF-β signaling and complement-tagged synapses then consolidate hyperexcitability over time. Within this cascade, cytokine/TLR signaling is particularly well characterized. Seizure activity rapidly upregulates IL-1β, TNF-α, and HMGB1, which in turn act via IL-1R, tumor necrosis factor receptor, and TLR4/RAGE to lower seizure threshold and amplify glial activation. Mechanistically, IL-1β enhances NMDA-receptor-mediated Ca²⁺ influx through Src-dependent phosphorylation of NR2 subunits in hippocampal neurons, thereby exacerbating excitotoxicity and promoting seizure propensity. HMGB1, released from injured neurons, engages TLR4 and RAGE on glia and neurons to propagate inflammatory signaling and network instability. These effects have been demonstrated across models and supported by patient data, reinforcing their translational relevance [2,10,11].

Complement pathways are likewise implicated. Components of the classical cascade, notably C1q and C3, are upregulated after status epilepticus and in resected human temporal lobe epilepsy (TLE) tissue, where they localize to microglia, astrocytes, and neurons and correlate with synaptic loss and gliosis. Complement-driven maladaptive synaptic pruning and C5aR1-mediated inflammation appear to contribute to hyperexcitability; pharmacologic C5aR1 antagonism (e.g., PMX53-class compounds) increases seizure threshold and reduces neuronal degeneration in multiple rodent models, identifying a tractable anti-inflammatory and potentially anticonvulsant target for translation [2,11].

Sustained BBB dysfunction further entrenches epileptogenesis. Inflammatory mediators and matrix metalloproteinases (MMPs) compromise endothelial tight junctions, increase leukocyte adhesion and diapedesis, and permit serum proteins to enter the parenchyma. A key downstream event is albumin uptake by astrocytes with activation of TGF-β/activin receptor-like kinase 5 (ALK5) signaling, downregulation of Kir4.1 channels, impairing K⁺ buffering, and promotion of excitatory synaptogenesis changes that foster a proepileptogenic microenvironment; blocking pathological TGF-β signaling mitigates these effects in experimental systems [2,9,11].

Crucially, these inflammatory and barrier mechanisms can persist long after the inciting insult, establishing a chronic inflammatory state that primes the brain for spontaneous seizures and resistance to conventional ASMs. Collectively, this framework supports therapeutic strategies that aim to modify disease biology, for example, targeting the IL-1/IL-6 axis and TLR/HMGB1 signaling, inhibiting complement, and stabilizing the BBB, rather than relying solely on symptomatic suppression of neuronal firing. As mechanistic biomarkers and patient-selection tools improve, these approaches may be deployable not only in overt autoimmune encephalitides but also across broader DRE phenotypes where neuroinflammation is a sustaining driver.

## Review

Pathophysiology of neuroinflammation in epileptogenesis

Neuroinflammation is increasingly recognized as a central mechanism in epileptogenesis. Whereas acute inflammatory responses are adaptive and self-limited, failure of resolution creates a chronic, dysregulated milieu that perpetuates neuronal injury, synaptic dysfunction, and seizure propensity. Convergent clinical and experimental work supports this framework and links inflammatory signaling to drug resistance and network remodeling in epilepsy [11].

Epileptogenesis can be initiated by diverse insults, including traumatic brain injury, CNS infection, ischemia or stroke, prolonged seizures/status epilepticus, and, in some syndromes, pathogenic genetic variants (e.g., SCN1A, PCDH19), that trigger release of danger-associated molecular patterns (DAMPs) such as ATP, heat-shock proteins, HMGB1, and extracellular DNA. DAMPs activate pattern-recognition receptors (PRRs) on microglia and astrocytes, notably TLRs and RAGE, initiating innate immune signaling that lowers seizure threshold and promotes network instability [11,12].

Upon PRR engagement, microglia (the resident immune cells) and astrocytes shift to reactive states and release proinflammatory mediators, including IL-1β, TNF-α, and IL-6, along with chemokines such as MCP-1 and CXCL10 and reactive oxygen/nitrogen species. These cascades form a feedforward loop that perturbs neuron-glia communication and augments excitability. Mechanistically, IL-1β enhances NMDA-receptor-mediated Ca²⁺ influx and excitotoxicity, while HMGB1 released from injured neurons engages TLR4/RAGE on glia and neurons to amplify epileptogenic signaling; experimental blockade of IL-1β or TLR4 reduces seizure severity and epileptogenesis in models, and anakinra (IL-1R antagonist) shows translational signals in selected inflammatory epilepsies [11,13].

A pivotal downstream consequence is BBB dysfunction. Inflammatory mediators and matrix metalloproteinases (MMP-2/9) degrade tight-junction proteins and activate endothelium, increasing leukocyte trafficking and parenchymal entry of serum proteins. Albumin extravasation and uptake by astrocytes activate TGF-β/ALK5 signaling, downregulate Kir4.1 channels, impair K⁺ buffering, and drive excitatory synaptogenesis, changes that sustain hyperexcitability; TGF-β pathway activation after BBB breach has been causally linked to epileptiform activity in experimental systems [14].

Chronic neuroinflammation then reshapes circuit architecture. Aberrant mossy-fiber sprouting, loss of inhibitory tone, and excess glutamatergic transmission are reinforced by complement-mediated synaptic pruning. C1q and C3, initiators of the classical pathway, localize to epileptic human cortex and increase after status epilepticus in models, correlating with synaptic loss and gliosis. Manipulation of the C3/C3aR axis modifies neurodegeneration and seizure phenotypes in preclinical studies. Together, these data implicate complement-glia crosstalk as a driver of persistent network hyperexcitability long after the initial insult [11,14].

Complement pathways in epilepsy

The complement system, classical (C1q→C4/C2→C3), lectin, and alternative pathways converging on C3 and the terminal C5b-9 membrane attack complex (MAC), is a multifunctional innate effector in the brain, regulating synaptic refinement, chemotaxis, and, at extremes, cytolysis. In epilepsy, classical-pathway dysregulation is most consistently implicated across human tissue and animal models.

Classical-Pathway Initiation (C1q)

C1q tags synapses for microglial pruning during development; in epilepsy, sustained or mislocalized C1q activity appears to contribute to maladaptive synaptic elimination. Increased C1q (and downstream C3) has been documented in resected human TLE cortex and after status epilepticus in rodents, in association with dendritic/synaptic pathology [15].

Amplification Node (C3/C3aR)

Astrocytes are a major source of C3 in neuroinflammation. C3/C3aR signaling strengthens microglia-astrocyte communication, promotes gliosis, and exacerbates neuronal injury; genetic deletion or pharmacologic depletion of C3 mitigates status-epilepticus-related neurodegeneration and memory deficits in models, highlighting this axis as a tractable target [16].

Effector Node (C5a/C5aR1)

C5aR1 is upregulated after seizures and drives potent proinflammatory signaling. Antagonism with PMX53-class compounds raises seizure threshold and reduces neurodegeneration and mortality in rodent models, an anticonvulsant/anti-inflammatory mechanism with translational potential.

Terminal Pathway (MAC) and CD59 Restraint

Excess MAC can directly injure neurons and glia. In Rasmussen syndrome, CSF studies demonstrate elevated iC3b, C5a, and soluble MAC, and implicate inadequate MAC control (CD59) in worse seizure profiles, strengthening the human link between complement activation and severe epilepsy. CD59 is the endogenous membrane inhibitor that blocks MAC assembly on host cells; deficits in CD59 or its function increase vulnerability to complement-mediated damage [17-19]. Overall, the epilepsy literature supports a node-based model in which aberrant C1q/C3 activity remodels synapses and glia, C5aR1 signaling amplifies inflammation and excitability, and terminal-pathway overactivity risks direct cytotoxicity, each providing a mechanistic foothold for therapy.

Emerging therapeutics targeting complement pathways

This growing understanding of the complement system's role has spurred the development of novel therapeutic strategies aimed at interrupting this destructive cascade. Several promising agents, summarized in Table 1, are currently under investigation. ANX005 is a monoclonal antibody designed to target C1q. Currently, in advanced clinical trials for other neuroinflammatory disorders, it has demonstrated efficacy in reducing complement-mediated pathology and is a prime candidate for repurposing in epilepsy [20]. Another agent, PMX53, is a selective C5aR antagonist that has already demonstrated convincing anticonvulsant and neuroprotective effects in preclinical epilepsy models [21]. Finally, CD59 mimetics are a class of agents designed to inhibit the formation of the MAC. By mimicking the natural inhibitor, these therapeutics may protect neurons from complement-mediated lysis and are under specific investigation for AAEs [22].

**Table 1:** Complement targets in epilepsy GBS: Guillain-Barré syndrome; MAC: membrane attack complex; RS: Rasmussen syndrome Source: [15-20]

Target/component	Main function in epilepsy	Therapeutic agent	Trial status/notes
C1q	Synaptic pruning, inflammation	ANX005	Phase 3 in GBS; potential in epilepsy
C3/C3aR	Microglia-astrocyte crosstalk, neurodegeneration	C3 inhibitors (preclinical)	Preclinical, promising in animal models
C5aR	Proinflammatory signaling, neuronal injury	PMX53	Preclinical, effective in seizure models
MAC	Direct neuronal lysis	CD59 mimetics	Investigational, relevance in RS
C4	Synaptic pruning, immune activation	No direct agent	Pathogenic role, target for future studies

Immune checkpoints and CNS immune tone

Immune checkpoints are critical regulators of immune activation and tolerance, maintaining a balance between immune suppression and the clearance of autoantigens. In the CNS, dysregulation of these pathways can contribute to chronic neuroinflammation, autoimmunity, and potentially epileptogenesis (Table 2).

**Table 2:** Immune checkpoints in the CNS CNS: central nervous system; PD-1: programmed cell death protein 1; PD-L1: programmed death-ligand 1; CTLA-4: cytotoxic T-lymphocyte-associated antigen 4; TIGIT: T cell immunoreceptor with Ig and ITIM domains; NK: natural killer; IL-10: interleukin-10 Source: [21-23]

Checkpoint	Main role	CNS relevance in epilepsy
PD-1/PD-L1	Inhibits T cell activation, promotes immune tolerance	Upregulated in neuroinflammatory states; blockade can exacerbate autoimmunity and seizures
CTLA-4	Suppresses T cell responses, maintains self-tolerance	Expressed on T cells and NK cells; regulatory phenotype linked to reduced cytotoxicity and increased IL-10
TIGIT	Inhibits T and NK cell activity	High baseline cytotoxicity in NK cells; expression modulates immune response in CNS
LAG3	Regulates T cell activation and exhaustion	Associated with regulatory NK cell phenotype and impaired cytotoxic function

Immune checkpoint expression and function in epilepsy

Having established a framework where innate immune signaling and the complement system maintain a state of neuronal excitability, it is logical to next examine immune checkpoints. These are the inhibitory receptors that fine-tune immunity throughout the body and, we now know, also govern the delicate interplay between neurons and glial cells in the brain. The PD-1/PD-L1 interaction serves as the most thoroughly studied example. Its classic function is to curb the activity of T-cells, but PD-1 is also present on excitatory neurons within the hippocampus. In this neural context, PD-1/PD-L1 signaling directly regulates the cell's inherent excitability and its capacity for synaptic plasticity. When this pathway is experimentally disrupted, the result is increased neuronal firing and strengthened long-term potentiation (LTP). This provides a clear, causal connection between the activity level of this checkpoint and neuronal hyperexcitability. Beyond neurons, when astrocytes become reactive following brain injury, they show increased production of PD-L1. These astrocytes employ the PD-L1 pathway to actively reduce neuroinflammation in the living brain. Collectively, these findings indicate that checkpoint pathways function as native restraining mechanisms within the CNS, directly limiting both excessive excitability and inflammation, and are not merely peripheral regulators of immunity [23].

Clinical experience with cancer immune checkpoint inhibitors (ICIs) underscores the axis’s relevance to seizures. Systemic PD-1/PD-L1 or CTLA-4 blockade can precipitate immune-related encephalitis with seizures in a subset of patients, and contemporary series continue to define the spectrum of CNS immune toxicities associated with ICIs. While these events arise in a different therapeutic context, they illustrate that disinhibition of checkpoint signaling can lower the barrier to neuroinflammation and ictogenesis in humans, an important caution as epilepsy moves from descriptive to interventional checkpoint biology [23,24].

Whether checkpoint expression is uniformly “increased” on T and natural killer (NK) cells in epilepsy remains unclear and appears context-dependent. Small human studies in TLE show peripheral immune cell shifts consistent with chronic immune activation, and emerging epilepsy-specific work is probing PD-1-positive lymphocyte populations. However, robust, replicated datasets demonstrating a stereotyped increase of PD-1, CTLA-4, or lymphocyte activation gene 3 across epilepsy cohorts are still lacking. What is well established from broader neuroinflammatory and oncologic literature is that heightened checkpoint expression on lymphocytes often marks a regulatory/exhausted phenotype with greater IL-10 and reduced effector cytokines, but this phenotype does not generalize across all tissues and diseases. In the NK-cell compartment, for example, T cell immunoreceptor with Ig and ITIM domains (TIGIT) can associate with reduced cytotoxicity in some cancers, yet in glioblastoma, TIGIT⁺ NK cells can remain hyperfunctional or act through decoy-like mechanisms, underscoring that TIGIT biology depends on ligand availability and the surrounding cytokine milieu. In epilepsy, definitive NK/T-cell checkpoint phenotyping (blood vs. CSF vs. tissue) remains an open research need [24].

Translationally, these data point to measured strategies rather than wholesale checkpoint blockade. Given neuronal and astrocytic engagement of PD-1/PD-L1 within epileptogenic circuits, brain-targeted augmentation of inhibitory checkpoint tone, for instance, approaches that enhance PD-1 signaling locally, may offer a way to dampen excitability and inflammation without provoking the systemic immune activation seen with oncology-style ICIs. Any such approach will require biomarker-guided selection and careful monitoring, but it aligns conceptually with the broader shift in epilepsy therapeutics from symptomatic suppression toward mechanism-directed disease modification [24,25].

Balancing immune suppression and autoantigen clearance

The activation of immune checkpoints presents a complex biological balance, while preventing excessive immune-mediated damage to healthy neural tissue, it may simultaneously hinder the clearance of pathogenic autoantigens. This impaired clearance mechanism can perpetuate chronic inflammation and increase seizure susceptibility [26]. The delicate equilibrium required in modulating these pathways is further emphasized by observations from cancer immunotherapy, where checkpoint inhibition has been shown to trigger CNS autoimmunity, including seizure activity and encephalitis [26,27].

Therapeutic insights

Several therapeutic strategies are emerging to target immune checkpoint pathways in epilepsy. Enhancement of PD-1/PD-L1 signaling through PD-L1 agonism represents a promising approach to suppress neuroinflammation and potentially reduce seizure risk. Abatacept (CTLA-4-Ig), a selective costimulation modulator currently used in autoimmune diseases that functions by blocking T cell activation, shows potential for application in epilepsy cases characterized by immune dysregulation. Additionally, investigation continues into modulation of the TIGIT and LAG3 pathways for their roles in immune regulation and potential to restore CNS immune homeostasis [22].

Translational and clinical perspectives

The advancing understanding of neuroimmunology in epilepsy necessitates parallel developments in translational strategies, particularly in biomarker discovery, precision medicine, and clinical trial design. Targeting immune mechanisms offers a unique therapeutic opportunity not only for seizure management but also for disease course modification, especially in drug-resistant and autoimmune epilepsies.

The identification of reliable biomarkers remains essential for diagnosis, prognosis, and patient selection in immunotherapy trials. Several promising candidates have emerged, including IL-1β in CSF, where elevated levels associate with active inflammation, increased seizure frequency, and resistance to antiepileptic drugs. IL-1β reflects central cytokine activity and correlates with microglial activation [28]. Serum glial fibrillary acidic protein (GFAP), detectable with ultrasensitive assays such as Simoa, serves as a marker of astrocytic injury that correlates with disease severity and seizure burden, though it lacks specificity to epilepsy as it elevates in various neurodegenerative conditions [29]. Translocator protein positron-emission tomography (TSPO-PET) imaging enables noninvasive visualization of neuroinflammation by detecting translocator protein expression on activated microglia and astrocytes, potentially identifying inflammation in epileptogenic zones and predicting seizure onset and treatment response [30].

Precision targeting of immune pathways requires careful patient stratification based on clinical, genetic, and immunological profiles. Autoantibody panels detecting antibodies against targets such as NMDAR, LGI1, glutamic acid decarboxylase 65, and α-amino-3-hydroxy-5-methyl-4-isoxazole propionate receptor are critical for identifying autoimmune epilepsy that may respond better to immunomodulation than to standard ASMs. Human leukocyte antigen (HLA) typing identifies specific alleles, including HLA-DRB1*07:01, that associate with susceptibility to autoimmune seizures and hypersensitivity to certain drugs such as carbamazepine, informing both diagnostic and therapeutic decisions [31]. Clinical phenotyping recognizes that features, including early-onset seizures, resistance to ASMs, neuropsychiatric comorbidities, and CSF pleocytosis, increase the likelihood of immune-mediated epilepsy and should guide therapeutic strategy [32].

Traditional clinical trials in epilepsy primarily focus on seizure reduction as the primary endpoint. For immunotherapies, however, broader disease-modifying outcomes warrant consideration. Primary endpoints might include seizure freedom, changes in seizure severity scores, and time to disease progression. Secondary endpoints could encompass biomarker response, such as reduction in IL-1β or TSPO signal, MRI volumetrics, cognitive improvement, and resolution of inflammation (Table 3). Stratification criteria should incorporate antibody status, imaging evidence of inflammation, and BBB disruption. Furthermore, study designs must include sufficient longitudinal follow-up to capture potential delayed effects of immune modulation.

**Table 3:** Neuroinflammatory biomarkers and their clinical associations IL-1β: interleukin-1 beta; CSF: cerebrospinal fluid; CNS: central nervous system; GFAP: glial fibrillary acidic protein; TSPO: translocator protein; PET: positron-emission tomography; LGI1: Leucine-rich glioma-inactivated 1; NMDAR: N-methyl-D-aspartate receptor; AMPAR: α-amino-3-hydroxy-5-methyl-4-isoxazole propionate receptor; GAD65: glutamic acid decarboxylase 65; HLA: human leukocyte antigen Source: [29,30]

Biomarker	Source	Associated features	Clinical relevance
IL-1β	CSF	Seizure frequency, drug resistance	Marker of active CNS inflammation
GFAP	Serum	Astrocytic injury, seizure severity	Blood-based monitoring of glial damage
TSPO (via PET)	Brain imaging	Activated microglia in epileptic foci	Noninvasive detection of neuroinflammation
Autoantibodies	Serum/CSF	LGI1, NMDAR, AMPAR, GAD65	Diagnostic and prognostic in autoimmune epilepsy
HLA-DRB1*07:01	Genomic DNA	Drug sensitivity, autoimmune risk	Predictive biomarker for stratification

The comparative and synergistic strategies are displayed in Table 4.

**Table 4:** Comparative overview of immune-targeted approaches BBB: blood-brain barrier; PD-1: programmed cell death protein 1; PD-L1: programmed death-ligand 1; CTLA-4: cytotoxic T-lymphocyte-associated antigen 4; CNS: central nervous system; IL-1β: interleukin-1 beta Source: [29-32]

Strategy	Mechanism of action	Benefits	Risks/challenges
Complement inhibition	Blocks complement cascade (e.g., C1q, C3, and C5aR), reducing synaptic pruning, neuroinflammation, and neuronal injury	Reduces neuroinflammatory damage and synaptic loss; promising preclinical and early clinical data	Systemic inhibition may impair host defense and homeostasis; risk of infections; BBB penetration challenges
Checkpoint modulation	Modulates immune checkpoints (PD-1/PD-L1, CTLA-4, etc.) to restore immune balance and limit autoimmunity	May suppress chronic neuroinflammation and autoantigen-driven seizures; targets regulatory immune tone	Oversuppression may hinder necessary immune responses; excessive inhibition can trigger CNS autoimmunity
IL-1β blockade	Inhibits IL-1β signaling, dampening cytokine storm and downstream inflammatory cascades	Directly reduces seizure susceptibility and inflammation; well-tolerated in other diseases	May blunt protective immune responses; risk of infection; limited data in chronic epilepsy

Benefits and risks

Therapeutic strategies that modulate neuroinflammation in epilepsy promise disease modification beyond symptomatic seizure suppression, but the same pathways that shape excitability also underwrite host defense and immune homeostasis. Consequently, benefits must be weighed against predictable class-specific toxicities and managed with biomarker-guided selection, vaccination, and infectious-risk prophylaxis. Complement-directed therapies are attractive because they intersect key epileptogenic nodes: C1q/C3-mediated synaptic tagging and pruning, C5a-driven inflammatory signaling, and terminal membrane-attack complex injury. In preclinical models, antagonizing C5aR1 with PMX53 reduces seizure severity, neuronal degeneration, and mortality, consistent with C5aR1 upregulation after status epilepticus and positioning this receptor as an anticonvulsant/anti-inflammatory target with a potentially narrower immunosuppressive footprint than proximal complement blockade. Translationally, upstream inhibition of the classical pathway is now clinically credible: the C1q-neutralizing antibody ANX005 achieved positive results in a pivotal Phase 3 Guillain-Barré syndrome trial, demonstrating target engagement and neurological benefit in a human neuroinflammatory disease and supporting exploration in epilepsy, where classical-pathway activity is implicated. These benefits are counterbalanced by the infection risk that scales with the point of pathway blockade. Terminal and C3 inhibition carry a boxed warning for invasive disease due to encapsulated bacteria, especially *Neisseria meningitidis*, *Streptococcus pneumoniae*, and *Haemophilus influenzae *type b, and breakthrough meningococcal infections can occur despite vaccination, necessitating vaccine completion, vigilance for early symptoms, and, in selected settings, antibiotic prophylaxis at initiation. By contrast, receptor-level antagonism of C5aR1 (e.g., PMX53) has not been associated with the same magnitude of invasive encapsulated bacterial risk in early studies. However, epilepsy-specific clinical safety data remain limited, and careful surveillance is still required [31-33].

Checkpoint-axis modulation offers a mechanistically distinct route to dampen excitability and inflammation. The PD-1/PD-L1 pathway is now recognized within the CNS: PD-1 is expressed by hippocampal principal neurons, and PD-L1 derived from astrocytes constrains neuroinflammation after injury. These observations imply that augmenting checkpoint tone locally could restrain hyperexcitability and glial activation. However, experience from oncology with systemic checkpoint blockade underscores the converse risk: disinhibition of the same axis can precipitate immune-related encephalitis with seizures, highlighting a narrow therapeutic window and the importance of brain-targeted or perilesional strategies rather than systemic checkpoint manipulation. For CTLA-4-pathway augmentation (abatacept), infection risks are those typical of biologic immunomodulation, serious bacterial infections, caution with live vaccines, and potential hepatitis B reactivation mandating baseline screening and risk-stratified antiviral prophylaxis in accordance with contemporary hepatology guidelines [33].

Cytokine-level strategies, most prominently IL-1 blockade, have the advantage of acting at a convergence point of innate inflammatory signaling. In NORSE/FIRES, anakinra has been associated across multicenter series with clinical improvement and acceptable safety, and tocilizumab (IL-6R blockade) has shown benefit in refractory cases, including during the chronic phase, supporting the biological relevance of these nodes when inflammation is prominent. Yet these approaches carry class-specific liabilities: anakinra is linked to serious infection and neutropenia (especially when stacked with other biologics), while tocilizumab has a boxed warning for serious infections and requires monitoring for transaminase elevations, neutropenia, and thrombocytopenia; both drug classes necessitate hepatitis B virus (HBV) screening and, when indicated, prophylaxis because immunomodulation can precipitate reactivation. Importantly, while IL-1/IL-6 blockade can acutely suppress seizure-promoting inflammation, they may not fully reverse the upstream circuits and BBB pathology that sustain epileptogenesis, underscoring the rationale for node-matched combinations and for timing earlier in the disease course when feasible [32,33].

Because BBB disruption both initiates and perpetuates inflammatory epileptogenesis, interventions that normalize BBB signaling could be disease-modifying with comparatively modest systemic immunosuppression. Albumin extravasation activates astrocytic TGF-β/ALK5 signaling, downregulates Kir4.1, and promotes excitatory synaptogenesis; targeting this cascade, for example, with losartan, which antagonizes TGF-β signaling in experimental models, attenuates epileptogenesis driven by vascular injury and albumin exposure. These data identify the BBB-TGF-β axis as a plausible adjunct target with a distinct risk profile [33].

Across all classes, the principal risk of combination immunomodulation is additive immunosuppression. Clinical practice outside epilepsy informs a consistent mitigation bundle: complete age-appropriate and pathogen-specific vaccination before complement or cytokine inhibitors when possible; screen for hepatitis B with serologies and initiate nucleos(t)ide prophylaxis in at-risk patients; avoid live vaccines while on biologics; and institute *Pneumocystis jirovecii* pneumonia (PJP) when corticosteroids ≥20 mg prednisone-equivalent are used for greater than or equal to four weeks alongside another immunosuppressant. For complement inhibitors specifically, meningococcal, pneumococcal, and Hib vaccination should precede therapy, with recognition that vaccination does not abolish risk and that early antibiotics are warranted for febrile illness. Finally, trial design in epilepsy should incorporate infection-risk registries, functional immune readouts, and CNS-targeted delivery where feasible to capture benefits on seizure control and network remodeling while minimizing systemic immune costs.

Combined and synergistic therapeutic strategies

The multiplicity of inflammatory drivers in epileptogenesis argues for rational combinations that engage distinct nodes of the cascade while avoiding indiscriminate immunosuppression. In principle, pairing orthogonal mechanisms such as upstream inflammasome blockade with checkpoint-axis augmentation, or complement-pathway inhibition with IL-1 signaling antagonism can suppress feedforward neuroinflammation more completely than any single agent, permit dose-sparing, and reduce off-target toxicity. Preclinical data illustrate the plausibility of these pairings: inhibiting the NLRP3 inflammasome with agents such as MCC950 attenuates IL-1β-driven pathology and reduces seizure severity in rodent models, while C5aR1 antagonism (PMX53-class) independently raises seizure thresholds and limits neurodegeneration; taken together, these nodes bookend complementary segments of the innate cascade. Evidence from human neuroinflammation further supports checkpoint tone as a CNS brake: PD-1/PD-L1 signaling in hippocampal neurons and reactive astrocytes restrains excitability and glial activation, suggesting that local enhancement of this axis could synergize with inflammasome or complement inhibitors by damping residual inflammatory drive at the circuit level. Any move toward combinations must contend with the central clinical reason to combine immunomodulators: the same combinations can materially raise infection risk. This risk is not abstract. Agents acting proximally in complement (e.g., anti-C1q/C3) or at the terminal pathway increase susceptibility to encapsulated bacteria; for C5 inhibitors, the Centers for Disease Control and Prevention emphasizes that even fully vaccinated patients remain at substantial residual risk of invasive meningococcal disease, with cases reported from nongroupable *N. meningitidis*, and recommends vaccination per Advisory Committee on Immunization Practices plus consideration of antimicrobial prophylaxis and high vigilance for early symptoms. Downstream, stacking cytokine blockers (e.g., IL-1 or IL-6 inhibitors) with steroids or other immunosuppressants increases serious-infection rates and requires screening and monitoring for hepatitis B reactivation with guideline-concordant antiviral prophylaxis in at-risk patients. Contemporary gastroenterology guidance explicitly endorses HBV testing for anyone considered for immunosuppression and supports prophylaxis when risk is more than minimal or multiple low-risk drugs are combined. Across oncology and rheumatology experience, PJP emerges as a predictable opportunist when corticosteroids at ≥20 mg prednisone, equivalent for greater than or equal to four weeks, are combined with another immunosuppressive agent; expert guidance and recent reviews support trimethoprim-sulfamethoxazole prophylaxis in such settings [32,33].

These risks shape how combination strategies should be designed and deployed in epilepsy. In acute, florid neuroinflammation (NORSE/FIRES), short-course IL-1 or IL-6 blockade has shown clinical signals in series and case reports, and could serve as an induction layer that rapidly suppresses cytokine surges; subsequent maintenance might then rely on agents with narrower systemic immunosuppressive footprints, such as C5aR1 antagonists or BBB-focused approaches, to consolidate network stabilization while infection risk is deescalated. For combinations that include complement inhibitors, meningococcal (MenACWY and MenB), pneumococcal, and Hib vaccinations should be completed before therapy whenever feasible, with acknowledgment that vaccination does not abolish risk; clinicians should provide safety-net antibiotics promptly at the first sign of febrile illness. Where steroid-containing regimens are necessary alongside a biologic, PJP prophylaxis, HBV screening with preemptive antivirals when indicated, and avoidance of live vaccines during therapy are essential guardrails [33].

Taken together, synergy in epilepsy will likely depend less on piling on broad immunosuppression and more on node-matched pairing with clear time-phasing: extinguish the cytokine storm; then maintain control with pathways (e.g., C5aR1, PD-1/PD-L1 signaling augmentation, BBB-TGF-β modulation) that restrain excitability and glial activation with minimal systemic immune cost. Such designs should be biomarker-guided, explicitly incorporate infection-risk mitigation and vaccination plans from the outset, and include prospective capture of opportunistic infections and HBV outcomes alongside seizure metrics to ensure that gains in seizure control are not offset by avoidable harms.

Future directions

The growing recognition of neuroinflammation as a central player in epileptogenesis opens new avenues for disease-modifying therapies, yet several challenges remain. One key priority is to delineate the precise roles of individual immune cells, such as microglia, astrocytes, infiltrating T cells, and endothelial cells. Emerging technologies like single-cell RNA sequencing and spatial transcriptomics offer powerful tools to uncover cell-specific immune profiles across different stages of epilepsy, from initiation to chronic progression. These approaches can help identify pathogenic microglial subsets or T-cell populations that could be selectively targeted, minimizing off-target effects and preserving physiological immune surveillance.

In parallel, the development of reliable biomarkers is essential for guiding precision immunotherapy. Biomarkers such as CSF IL-1β, serum GFAP, and translocator protein (TSPO) expression detected via PET imaging are promising candidates. Their incorporation into clinical protocols can facilitate early diagnosis, predict seizure evolution, stratify patients for specific immunotherapies, and monitor treatment response in real time. However, further validation in large-scale, multicenter cohorts is necessary to standardize their clinical application. Moreover, point-of-care testing for autoantibodies, BBB integrity, and proinflammatory cytokines could support rapid clinical decision-making, especially in acute settings like autoimmune encephalitis or febrile infection-related epilepsy syndrome (FIRES). However, the application of antibody testing requires careful interpretation due to significant limitations, including the risk of false-positive results, which can lead to misdiagnosis and unnecessary treatment. The sensitivity and specificity vary considerably depending on the antigen target, the assay methodology (e.g., live cell-based assays are often superior to fixed assays or immunohistochemistry), and the sample source (with CSF often demonstrating higher specificity for intrathecally synthesized antibodies than serum). Therefore, these tools must be viewed as components of a comprehensive diagnostic framework that prioritizes clinical correlation.

Given the complexity of immune mechanisms involved in epilepsy, combination strategies are likely to offer superior efficacy compared to monotherapies. For example, co-targeting PD-L1 and IL-1β pathways could simultaneously dampen adaptive and innate immune responses, while pairing C1q inhibition with NLRP3 blockade may protect synapses and prevent inflammasome activation. Future clinical trials should therefore explore dual or triple immunomodulatory regimens with titrated dosing strategies tailored to biomarker profiles, thereby maximizing therapeutic benefit while minimizing immunosuppression-related risks.

Pediatric and genetic epilepsies represent another critical focus for translational research. Many childhood-onset epilepsies, including those associated with SCN1A mutations or postinfectious inflammatory triggers, display robust immune activation, indicating a potential role for early immune intervention. Tailoring immunotherapies based on the genetic and immunological phenotype of the patient could prevent the long-term cognitive and behavioral impairments often seen in these syndromes. Furthermore, the traditional design of epilepsy trials must evolve to accommodate the distinct nature of immune-based interventions. Rather than measuring only seizure frequency, new trials should consider broader endpoints such as cognitive function, inflammatory biomarker normalization, imaging markers, and overall quality of life. Adaptive trial designs that allow mid-course stratification based on biomarker changes may accelerate the development of personalized immunotherapies. Regulatory frameworks must also adapt to accommodate these novel endpoints and therapeutic mechanisms. A systems biology perspective is crucial to fully understand the neuroimmunology of epileptogenesis. Integrating computational modeling, artificial intelligence, and cross-disease comparisons (e.g., with multiple sclerosis or Alzheimer’s disease) can help generate predictive frameworks for disease course and treatment response. Ultimately, this multidimensional approach will usher in a new era of "precision immunoepileptology," in which seizures are not just suppressed but the brain's immune landscape is actively reshaped to restore neural function and prevent disease progression.

## Conclusions

Neuroinflammation is now established as a central mechanism in epilepsy, driving epileptogenesis and sustaining seizure activity through a self-amplifying cycle of immune activation. Key findings highlight the critical roles of complement-mediated synaptic pruning, dysregulated immune checkpoint expression, cytokine signaling, and BBB disruption in creating a hyperexcitable neural environment. These pathways represent promising therapeutic targets, with emerging evidence supporting the efficacy of complement inhibitors, cytokine blockers, and immune checkpoint modulators in modifying disease progression. The translation of these findings into clinical practice demands a precision medicine approach. Successful implementation will rely on advanced biomarker stratification-including neuroimaging, CSF analysis, and autoantibody profiling-to identify patients most likely to benefit from immunotherapeutic interventions. Future clinical trials must incorporate multidimensional endpoints that capture not only seizure reduction but also neuroinflammatory resolution and cognitive improvement. As research advances, combination therapies targeting multiple immune pathways simultaneously may offer the most effective strategy for achieving sustained seizure control and true disease modification. The ultimate goal remains the restoration of neural-immune homeostasis, moving beyond symptomatic treatment toward comprehensive therapeutic outcomes that include neuroprotection and functional recovery.
